# Insects' RNA Profiling Reveals Absence of “Hidden Break” in 28S Ribosomal RNA Molecule of Onion Thrips, *Thrips tabaci*

**DOI:** 10.1155/2015/965294

**Published:** 2015-02-12

**Authors:** Rosaline Wanjiru Macharia, Fidelis Levi Ombura, Erick Onyango Aroko

**Affiliations:** International Center for Insect Physiology and Ecology (icipe), P.O. Box 30772, Nairobi 00100, Kenya

## Abstract

With an exception of aphids, insects' 28S rRNA is thought to harbor a “hidden break” which cleaves under denaturing conditions to comigrate with 18S rRNA band to exhibit a degraded appearance on native agarose gels. The degraded appearance confounds determination of RNA integrity in laboratories that rely on gel electrophoresis. To provide guidelines for RNA profiles, RNA from five major insect orders, namely, Diptera, Hemiptera, Thysanoptera, Hymenoptera, and Lepidoptera, was compared under denaturing and nondenaturing conditions. This study confirmed that although present in most of insect's RNA, the “hidden break” is absent in the 28S rRNA of onion thrips,* Thrips tabaci*. On the other hand, presence of “hidden break” was depicted in whiteflies' 28S rRNA despite their evolutionary grouping under same order with aphids. Divergence of 28S rRNA sequences confirms variation of both size and composition of gap region among insect species. However, phylogeny reconstruction does not support speciation as a possible source of the hidden break in insect's 28S rRNA. In conclusion, we show that RNA from a given insect order does not conform to a particular banding profile and therefore this approach cannot be reliably used to characterize newly discovered species.

## 1. Introduction

Insects (Arthropoda: Insecta) constitute up to 55% of characterized species, playing different roles in the environment [1]. For instance, fruit flies, thrips, stem borers, and aphids are crop pests, tsetse flies, and mosquitoes transmit pathogens, while honey bees have direct economic benefits. Molecular characterization of insects involving RNA-Seq and quantitative RT-PCR employ RNA [1]. However, despite many years of research on insect rRNA, misinterpretation of their RNA profiles is common to molecular biologists, partly due to the variations in 28S rRNA thermolability in different insect species.

Success of downstream RNA application depends on the quality and integrity of the extracted RNA. Isolation of high quality RNA from biological samples is however challenged by the ubiquitous presence of ribonucleases (RNases) that rapidly degrade freshly prepared RNA [2]. This makes the assessment of RNA quality mandatory prior to its downstream application. The assessment usually relies heavily on the resolution of 18S and 28S rRNA bands on a gel, either through automated bioanalyzers [3] or denaturing agarose gel electrophoresis [4]. The bioanalyzer approach is more sensitive, but relatively costly, relegating most resource poor laboratories to the denaturing gel electrophoresis approach. It is therefore important to know how to accurately interpret RNA profiles in agarose gels. Under denaturing conditions, 28S rRNA from insects and most protostomes is characterized by its dissociation into two equally sized *α* and *β* subunits [5], which comigrates with the 18S rRNA to exhibit a single band profile. This dissociation of 28S rRNA molecule is attributed to the presence of an UAAU tract in their rRNA loop which acts as the cleavage site, also known as the “hidden break” [6]. The UAAU tract is located about 10 bases upstream of 5′ end of 28S *β* segment of 28S rRNA molecule. In addition, the presence of “hidden break” has also been reported in the 5.8S rRNA of Drosophila and in higher plant chloroplasts [7]. In contrast, the pea aphid 28S rRNA, just like deuterostomes 28S rRNA, does not harbor the “hidden break” but instead contains a GC-rich hairpin loop that lacks the UAAU tract [5, 7].

Efforts to understand molecular mechanisms involved in the introduction of the gap have been hindered by unavailability of 28S rRNA secondary structures and high conservation of sequences reported in the regions that flank the “hidden break.” This makes it difficult to compare the “hidden breaks" [7]. Nevertheless, an earlier study suggested that the hidden break is probably introduced into the polynucleotide chain during or after the processing of 28S rRNA from its precursor [5].

In the present study, banding profiles of RNA from five major insect orders considered of economic importance including Diptera, Hemiptera, Thysanoptera, Hymenoptera, and Lepidoptera were compared with an aim of providing guidelines to the appearance of RNA for insects belonging to these orders. Importantly, the absence of a hidden break was observed in the onion thrips,* Thrips tabaci*.

## 2. Materials and Methods

### 2.1. Insect Sampling

Representative insects for the five orders were obtained from insect colonies maintained at the* icipe* insectaries and screen houses. They included 150 aphids (Hemiptera);* Brevicoryne brassicae*, 60 mosquitoes (Diptera);* Anopheles arabiensis*, 20 tsetse flies (Diptera);* Glossina fuscipes fuscipes*, 16 honey bees (Hymenoptera);* Apis mellifera*, 200 whiteflies (Hemiptera); of Aleyrodidae family, six stem borers (Lepidoptera);* Chilo partellus*, four silkworms (Lepidoptera);* Bombyx mori*, 40 wasps (Hymenoptera);* Fopius arisanus*, 10 fruit flies (Diptera);* Dacus bivittatus* and 1000 thrips (Thysanoptera);* Thrips tabaci*. The sample size was informed by the size of the fly and experiments were done in replicates.

### 2.2. RNA Extraction

RNA from the insect samples was extracted using the Direct-zol RNA Mini Prep Kit (Zymo Research Corporation, Irvin, CA, USA), following the manufacturer's instructions. Fresh whole insect flies (≤50 mg) were homogenized in 500 *μ*L of TRI-reagent and centrifuged in an Eppendorf AG 5417R centrifuge (Hamburg, Germany) at 12,000 ×g for 10 min. The supernatant was transferred into a new tube followed by addition of one volume of absolute ethanol into the supernatant. After vortexing, 700 *μ*L of the mixture was transferred into a Zymo-Spin IIC Column in a collection tube, centrifuged at 12,000 ×g for 1 min and the flow through discarded. The spin column was transferred into a new collection tube and 400 *μ*L Direct-zol RNA prewash solution was added and centrifuged at 12,000 ×g for 1 min. The flow through was discarded and this step was repeated. Into the column, 700 *μ*L RNA wash buffer was added and centrifuged at 12,000 ×g for 1 min. The flow through was discarded and the column spun again at 12,000 ×g for 2 min to completely remove any residual wash buffer. The column was then transferred into an RNase-free 1.5 mL Eppendorf tube and 25 *μ*L of DNase/RNase-free water was added onto the column matrix, centrifuged at maximum speed for 1 min for RNA elution. The eluted RNA was immediately used for downstream processes.

### 2.3. Determination of RNA Yield and Purity

The quantity and quality of the extracted RNA yield were determined using a Nanodrop (ND) 2000c Spectrophotometer, (Thermo Scientific, Wilmington, USA) by measuring optical density of 2 *μ*L of extracted RNA at A_260 nm_ and A_280 nm_, respectively. Any sample with A_260 nm_/A_280 nm_ ratio below 1.7 was discarded.

### 2.4. Denaturing Agarose Gel Electrophoresis

A 1.2% formaldehyde agarose (FA) gel was prepared by mixing 1.2 g of agarose with 10 mL of 10X FA gel buffer (200 mM MOPS, 50 mM sodium acetate, 10 mM EDTA, pH 7.0). This was topped to 100 mL using RNase-free water. The mixture was heated to melt the agarose and cooled to approximately 65°C, after which 1.8 mL of 37% 12.3 M formaldehyde and 6 *μ*L of 10 mg/mL ethidium bromide were added. The mixture was thoroughly but gently mixed, then poured onto a casting tray, and left to polymerize under a laminar flow chemical hood.

The RNA samples were mixed with 1 volume of 5X RNA loading dye prepared as per the Qiagen protocol [8]. Sixteen *μ*L of saturated aqueous bromophenol blue solution was mixed with 80 *μ*L of 500 mM EDTA of pH 8.0, 720 *μ*L of 37% 12.3 M formaldehyde, 2 mL of 100% glycerol, 3.084 mL formamide, and 4 mL of 10X FA gel buffer and topped up 10 mL with RNase-free water. One volume of the dye was added to three volumes of RNA sample and mixed by pipetting. The sample-dye mixture was down spun and incubated at 65°C for 5 min, then chilled on ice, and immediately loaded onto the FA gel.

The gel was run at 70 V in the 1X FA gel running buffer (100 mL of 10X FA gel buffer, 20 mL of 37% 12.3 M formaldehyde, and 880 mL RNase-free water) for 1 hour followed by visualization of the gel under UV illumination and analyzed using KODAK Gel Logic 200 Imaging System (Raytest GmbH, Straubenhardt).

### 2.5. Nondenaturing Agarose Gel Electrophoresis

A 1.2% agarose gel was prepared by adding 1.2 g of agarose into 100 mL of 1X TAE buffer and heating to dissolve. Six *μ*L of 10 mg/mL ethidium bromide was added to the mixture and the sample RNA then mixed with a non-denaturing 6X DNA loading dye and the samples loaded onto the gel, alongside a molecular weight DNA marker (O'GeneRuler 1 Kb plus DNA Ladder). Electrophoresis, visualization, and gel image analysis were carried out as outlined above.

### 2.6. Multiple Sequence Alignment, Phylogeny Reconstruction, and Secondary Structure Determination

Representative 28S rDNA sequences for the studied insect orders were obtained from GenBank [9] (Table 2). In case of species lacking sequences from the database, their sequences were substituted with those of closely related species. The 28S rDNA sequences of* G. fuscipes fuscipes* and* Brevicoryne brassicae* were substituted with* G. morsitans morsitans* and* A. glycine*, respectively. No relative sequences were recovered to represent* Dacus bivittatus* and* Chilo partellus* in phylogeny reconstruction. Multiple sequence alignment was performed using Muscle version 3.6 [10] and a phylogenetic tree reconstructed using PhyML [11]. General time reversible (GTR) model for substitution was applied with 100 bootstrap replicates. The resulting tree was rendered using fig tree [12].

A sequence containing mapped hidden break and its flanking regions in* B. mori* [7] was aligned and compared against those of* G. morsitans morsitans*,* T. knoxi*, and* A. glycine*. The species were chosen as representatives for presence and absence of “hidden break” as observed in the denatured gel.* T. knoxi* sequence was used in place of* T. tabaci* based on its length. Secondary structures of these regions were determined using “The Predict a Secondary Structure server” at http://rna.urmc.rochester.edu/RNAstructureWeb/Servers/Predict1/Predict1.html which combines four prediction algorithms. Structures with maximum free energies were adopted for comparison and mapping of the hidden break regions.

## 3. Results

Purity and quantity of extracted RNA were determined using a Nanodrop. Observed absorbance (260/280) ratio for the samples was above 2.0 (Table 1), which is the recommended range for pure RNA [8]. Concentration of all samples was >350 ng/*μ*L which was high enough to be viewed under gel electrophoresis.

**Figure 1:**
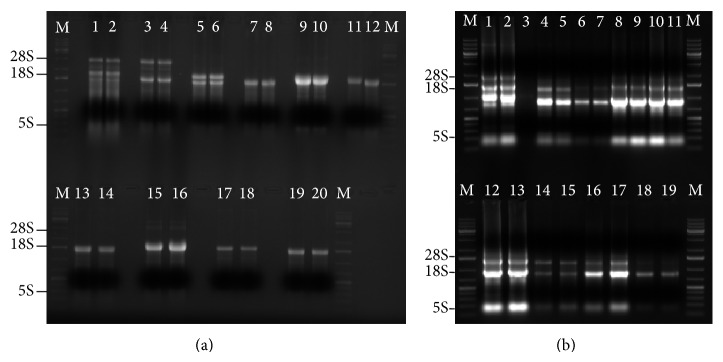
Gel images showing the banding profiles of the various insect RNA sampled. (a) Lane M: O'GeneRuler 1 kb DNA Plus (Thermo Scientific), Lanes 1 and 2: aphids, Lanes 3 and 4: thrips, Lanes 5 and 6: whiteflies, Lanes 7 and 8: mosquitoes, Lanes 9 and 10: tsetse flies, Lanes 11 and 12: fruit flies, Lanes 13 and 14: stem borer, Lanes 15 and 16: silk worm, Lanes 17 and 18: wasps, and Lanes 19 and 20: bees shows appearance of RNA on 1.2% formaldehyde agarose (FA) gel. On the other hand, (b) shows RNA appearance on 1.2% agarose non-denaturing gel; Lanes 1 and 2: aphids, Lane 3: whiteflies, Lanes 4 and 5: mosquitoes, Lanes 6 and 7: tsetse flies, Lanes 8 and 9: fruit flies, Lanes 10 and 11: stem borer, Lanes 12 and 13: silk worm, Lanes 14 and 15: bees, Lanes 16 and 17: wasps, and Lanes 18 and 19: thrips.

RNA profiles were visualized by both denaturing and non-denaturing agarose gel electrophoresis. Apart from aphids,* Brevicoryne brassicae* and their close “cousins”* Thrips tabaci*, other insects' RNA exhibited single banding profiles under denaturing condition (Figure 1(a)). On the other hand, under non-denaturing gel electrophoresis, all RNA samples appeared degraded (with 18S rRNA band showing higher intensity as compared to 28S rRNA band) (Figure 1(b)).

### 3.1. Sequence Homology and Phylogeny Reconstruction of 28S rRNAs

**Figure 2:**
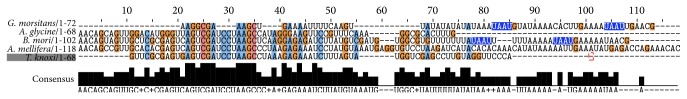
Multiple alignment of gap region of* B. mori* 28S rRNA sequence against corresponding region in* G. morsitans morsitans*,* A. mellifera*,* A. glycine*, and* T. knoxi*. Dots indicate gaps introduced during alignment to maximize homology. UAAU highlighted in blue forms the “hidden break” in rRNA sequences from different organisms.

Sequence homology of the “hidden break” and its flanking regions was assessed through multiple alignment and visualized in Jalview [13]. The rRNA alignment shows high variability across the insect species with sequences of* A. mellifera, G. morsitans morsitans,* and* B. mori* sharing more nucleotides compared to those of* T. knoxi* and* A. glycine* (Figure 2).

**Figure 3:**
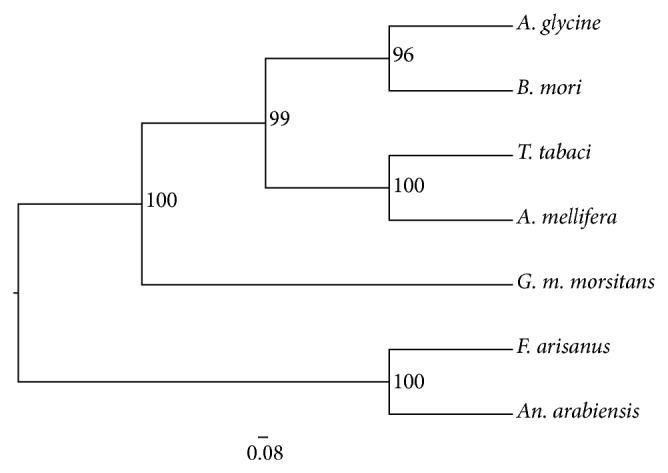
Amidpoint rooted maximum likelihood generated from 28S rRNA sequences for representative species in five major insect orders. The cladogram was generated using PhyML with 100 bootstrap replicates. Node labels show support bootstrap values.

GC content of the region around the gap region was assessed using EMBOSS geecee tool http://emboss.bioinformatics.nl/cgi-bin/emboss/geecee. Like* A. glycine* (49%),* T. knoxi* variable region sequence recorded a higher GC composition (47%) compared to that of* A. mellifera* (39%),* B. mori* (35%), and* G. m. morsitans* (22%). Evolutionary relatedness of 28S rRNA molecules of the studied insects was assessed by constructing a phylogenetic tree of their 28S rRNA sequences or of their close relatives in case of missing sequences. The resulting tree with bootstrap values is shown in Figure 3.

### 3.2. Model Secondary Structure of Thrips' 28S rRNA Variable Region

**Figure 4:**
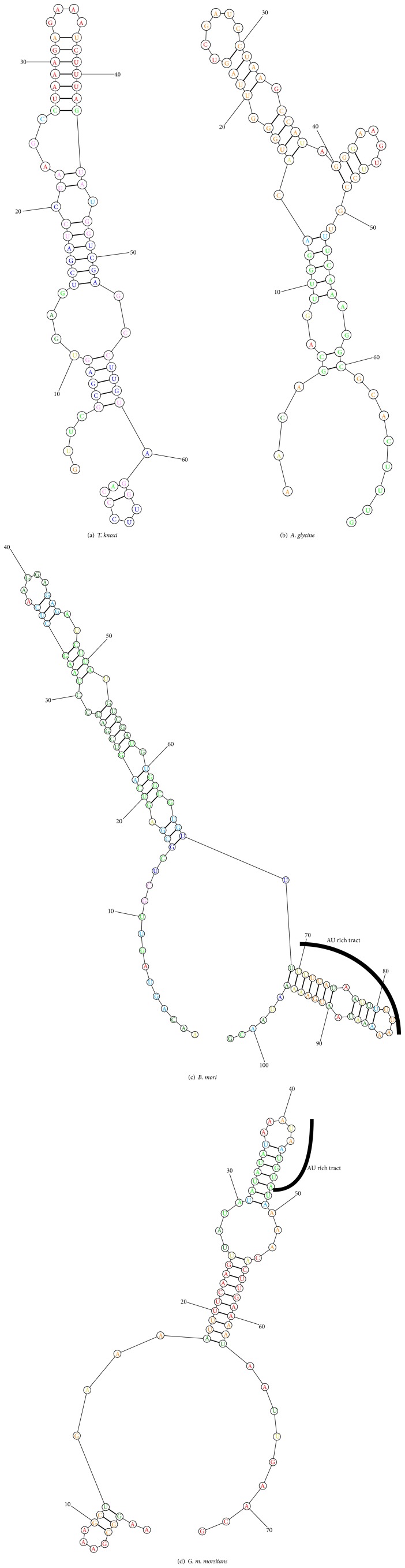
Comparison of proposed secondary structures for four insects' gap region of 28S rRNA sequences. The AU rich tract that forms the “hidden break” is indicated using an arc.

Possible secondary structure of thrips' gap region and its flanking region was generated using* T. knoxi* sequence and compared against that of* B. mori*,* G. morsitans morsitans,* and* A. glycine* (Figures 4(a)–4(d)).

## 4. Discussion

Earlier studies on insect RNA have reported presence of a “hidden break” that disintegrates under denaturing conditions in all insects except aphids [5–7, 14]. Molecular mechanism behind introduction of this hidden break remains unclear. However, its introduction has been attributed to postprocessing of the rRNA which forms a UAAU tract downstream of the *β* segment of 28S rRNA. Disintegration of 28S rRNA molecule at the hidden break UAAU tract is induced by heat denaturation that breaks hydrogen bonds joining the *α* and *β* molecules to yield a single-banded profile for majority of insects [14]. No specific buffer has been reported to affect this dissociation. Rather, heating of the RNA has been shown to yield similar results both under bioanalyzer analysis and on native agarose gels [14]. Results of this study confirm presence of “hidden break” in majority of the insects and also reveal the absence of a “hidden break” in the onion thrips,* Thrips tabaci*. Eight of the ten insects sampled here exhibit single banding profiles after denaturation (Figure 1(a)), which supports earlier findings by Lehrach et al. [4] and Winnebeck et al. [14]. Two clear bands (*α* and *β* molecules) that are seen in case of whiteflies (Figure 1(a), Lane 3) appear to comigrate closely with the 18S rRNA molecule. Their molecular size suggests dissociation of their 28S rRNA despite being in the same order with aphids. This scenario is similar to that of testis' rRNA from rodents of genus* Ctenomys* which exhibits the presence of “hidden break”, yielding a 2.6 Kb band and a 1.9 Kb band [15]. This could mean that evolution has got no role to play in introduction of hidden break in insect's RNA given that whiteflies and aphids are closely related evolutionarily.

In addition to aphids' RNA (Figure 1(a), Lanes 1 and 2) an intact band for thrips' 28S rRNA was observed which could imply that this molecule does not contain a “hidden break” (Figure 1(a), Lanes 3 and 4). This observation was further strengthened by comparative analysis of possible secondary structures of variable regions that aligned to* B. mori's* variable region. Structures predicted from* T. knoxi* and* A. glycine* did not contain the AU rich region but rather showed high GC content of 47% and 49%, respectively. Nevertheless, phylogenetic analysis of their sequences (Figure 3) did not support this observation. Instead, divergence was observed between their 28S rRNA sequences. On the other hand, structures of variable regions derived from* G. m. morsitans* and* B. mori* displayed an AU rich tract and both showed low GC composition (22% and 35%, resp.). In conclusion, we report absence of “hidden break” in one more insect, thrips, and suggest that the banding profile of all other insects may not always conform to a single band under denaturing conditions; thus RNA profiling should not be used as a means of characterizing newly discovered species.

## Figures and Tables

**Table 1:** Nanodrop readings for RNA samples: *Brevicoryne brassicae*, *Thrips tabaci*, whiteflies (Aleyrodidae), *Anopheles arabiensis*, *Glossina fuscipes fuscipes*, *Dacus bivittatus*, *Chilo partellus*, *Bombyx mori*, *Apis mellifera*, and *Fopius arisanus*.

Species name	Concentration (ng/*µ*L)	A_260/280 nm_
*Brevicoryne brassicae *	1794.8	2.13
*Brevicoryne brassicae *	1854.3	2.13
*Thrips tabaci *	1086.1	2.16
*Thrips tabaci *	1061.9	2.15
*Aleyrodidae *	647.1	2.06
*Aleyrodidae *	654.8	2.13
*Anopheles arabiensis *	478.0	2.03
*Anopheles arabiensis *	423.7	2.20
*Glossina fuscipes fuscipes *	1248.6	2.03
*Glossina fuscipes fuscipes *	1548.5	2.33
*Dacus bivittatus *	419.3	2.17
*Dacus bivittatus *	496.7	2.21
*Chilo partellus *	991.5	2.01
*Chilo partellus *	787.6	2.05
*Bombyx mori *	1177.2	1.92
*Bombyx mori *	1061.7	2.04
*Apis mellifera *	868.8	2.22
*Apis mellifera *	818.0	2.21
*Fopius arisanus *	448.9	2.09
*Fopius arisanus *	374.1	2.12

**Table 2:** Species names of 28S rDNA sequences used in comparative analysis alongside associated GenBank IDs, percentage GC content, and length in base pairs.

Species name	GenBank sequence ID	% GC content	Length (bp)
*An*. *arabiensis *	AF417812.1	54	527
*T*. *knoxi *	KC513119.1	55	1893
*T*. *tabaci *	AY523392.1	50	678
*F*. *arisanus *	JN640572.1	42	620
*A*. *mellifera *	AJ302936.1	56	2748
*B*. *mori *	AY038991.1	52	1192
*G*. *morsitans morsitans *	KC177834.1	39	2165
*A*. *glycine *	JQ259057	51	3547
